# Epidemiology of complex regional pain syndrome in Korea: An electronic population health data study

**DOI:** 10.1371/journal.pone.0198147

**Published:** 2018-06-04

**Authors:** Hyungtae Kim, Cheol-Hyeong Lee, Sung-Hun Kim, Yeon-Dong Kim

**Affiliations:** 1 Department of Anesthesiology and Pain Medicine, Presbyterian Medical Center, Jeonju, Republic of Korea; 2 Department of Anesthesiology and Pain Medicine, Wonkwang University College of Medicine, Iksan, Republic of Korea; 3 Wonkwang Institute of Science, Wonkwang University College of Medicine, Iksan, Republic of Korea; BG Trauma Center Ludwigshafen, GERMANY

## Abstract

Chronic regional pain syndrome (CRPS) is an inflammatory and neuropathic pain disorder characterized by the involvement of the autonomic nervous system with sensory, autonomic, motor, skin, and bone changes. At present, universally accepted consensus criteria for CRPS are not yet established, despite the diagnostic criteria proposed by the International Association for the Study of Pain (IASP). Various hypotheses for the pathophysiology of CRPS have been proposed; as a result, current therapeutic modalities are varied. General epidemiological data on CRPS are necessary for effective management. However, recent data on the epidemiology of CRPS in Korea are scarce. The aim of this study was to evaluate the incidence and other epidemiological features of CRPS in the general population in Korea. In this study on the epidemiology of CRPS in Korea, population-based medical data acquired from 51,448,491 subscribers to the National Health Insurance Service (NHIS) from 2011 to 2015 were analyzed, including the incidence, distribution by the CRPS type, regional distribution, monthly distribution, medical costs, and healthcare resource-utilization. The findings indicated that the incidence of CRPS in Korea was 29.0 per 100,000 person-years in 2015 and was correlated with patient age and sex. CRPS types included type I (63%) and type II (37%); moreover, the number of individuals with CRPS I have shown a growing trend since 2011. There was no monthly distribution, but there was regional variation according to the province. The medical departments managing CRPS I the most were orthopedics, internal medicine, anesthesiology and pain medicine, in order; however, patients with CRPS spent more money per visit in the departments of rehabilitation medicine, and anesthesiology and pain medicine. The incidence rate of CRPS in Korea was 29.0 per 100,000 person-years with an increasing trend, which was correlated with patient age in the 70s and female sex. CRPS type I was more common than CRPS type II; in addition, constant increase in medical expenses, regional imbalance, and differences in medical expense among medical specialties should be considered for early management of patients to reduce the disease burden in Korea. Sharing of knowledge about the diagnostic criteria of CRPS are also needed.

## Introduction

Complex regional pain syndrome (CRPS) is a disease with clinical features that include excruciating pain, sensory and vasomotor changes, and impaired motor function [1]. The International Association for the Study of Pain (IASP) defined it as various painful conditions following injury that appears regionally with predominantly late-onset abnormality, which exceeds the expected clinical course of the inciting event in magnitude and duration, often results in significant impairment of motor function, and shows variable progression over time [2]. CRPS usually develops after relatively mild trauma, but its intensity and duration are highly variable [3]. CRPS is classified into two types: CRPS type I (reflex sympathetic dystrophy) is thought to occur following minor injury or fractures, and CRPS type II (causalgia) develops after major peripheral nerve injury. In patients with CRPS, the condition is distinguished by the occurrence of a non-detectable nerve lesion, compared to the presence of an identified nerve lesion in CRPS II [4].

The cause of CRPS may be explained by neurological alteration; however, the definite etiology or pathogenesis of the disease is not completely understood. The symptoms of CRPS can vary with mild to severe impact on activities of daily living. Among the several diagnostic criteria, IASP criteria are commonly utilized for diagnosis of CRPS; however, there is still no consensus on the definition of CRPS [5]. Due to a variety of symptoms and available treatment options, patients with CRPS are managed using various clinical modalities across several medical fields. Moreover, despite reports of various therapeutic modalities and guidelines, the efficacy of treatment is currently unclear. In order to establish the effective treatment method, fundamental epidemiologic data are essential.

Epidemiology study of CRPS in the general population in Korea has not yet been conducted. In this study, we aimed to evaluate the epidemiological features of CRPS in Korea.

## Materials and methods

The institutional review board of our university hospital approved this study (approval number WKUH 201602-HRE-014). All Koreans (2015 population 51,529,338) have been mandatorily required to subscribe to the National Health Insurance Service (NHIS) since 1989. NHIS is the only public and state-run medical insurance system in Korea, and the numbers of subscribers of NHIS are estimated to be nearly the same as the number of Korean nationals. The insurance service is based on the residential status that reflects the prevalence of a disease entity by the region. All demographic data including age, sex, and address are collected by the NHIS based on the patients’ Korean National Identification (ID) numbers. The Health Insurance Review and Assessment Service (HIRA) is a quasi-government agency developed for the purposes of medical billing and adequacy of reimbursement under the Ministry of Health and Welfare of Korea. All data of medical proceedings, such as diagnosis, physical and laboratory examination, treatment, prescription and hospitalization by the hospital or clinic, are recorded precisely in a computerized database along with the individual Korean National ID number. The data base is updated on issuance of the proceedings and the stored data are accessible for further analyses. Therefore, the incidence rate, monthly variation, regional distribution, and utilization of healthcare resources of CRPS can be deduced from the data of 51,529,338 subscribers of NHIS from 2011 to 2015.

The cases of CRPS were calculated through the results obtained from a database search for the HIRA code of CRPS (M8900) which was assigned based upon the International Classification of Diseases (ICD)-9 code by the World Health Organization. The data from HIRA included the medical records of patients who received medical service with insurance coverage from January 1^st^, 2011 to December 31^st^, 2015. CRPS is classified as CRPS type 1 or type 2 by the NHIS, and type 1 is further classified according to the injured part of the body, such as shoulder, elbow and wrist, hand, pelvis and thigh, lower leg, ankle and foot, multiple sites and other sites (non-specified sites). Data from patients with overlapping symptoms were excluded from the analysis. Data were fully anonymized before accessed. The regional distribution was based on the national residence registration data from the Korean Statistical Information System of Statistics. We analyzed the incidence, seasonal variation, regional distribution, medical costs, and use of healthcare resources by the medical specialty associated with CRPS. All statistical analyses were conducted using SPSS, version 20.0 (IBM SPSS, Inc., Chicago, IL, USA). All variables are described as number or percentage.

## Results

### 1. Incidence of CRPS from 2011 to 2015

**Fig 1:**
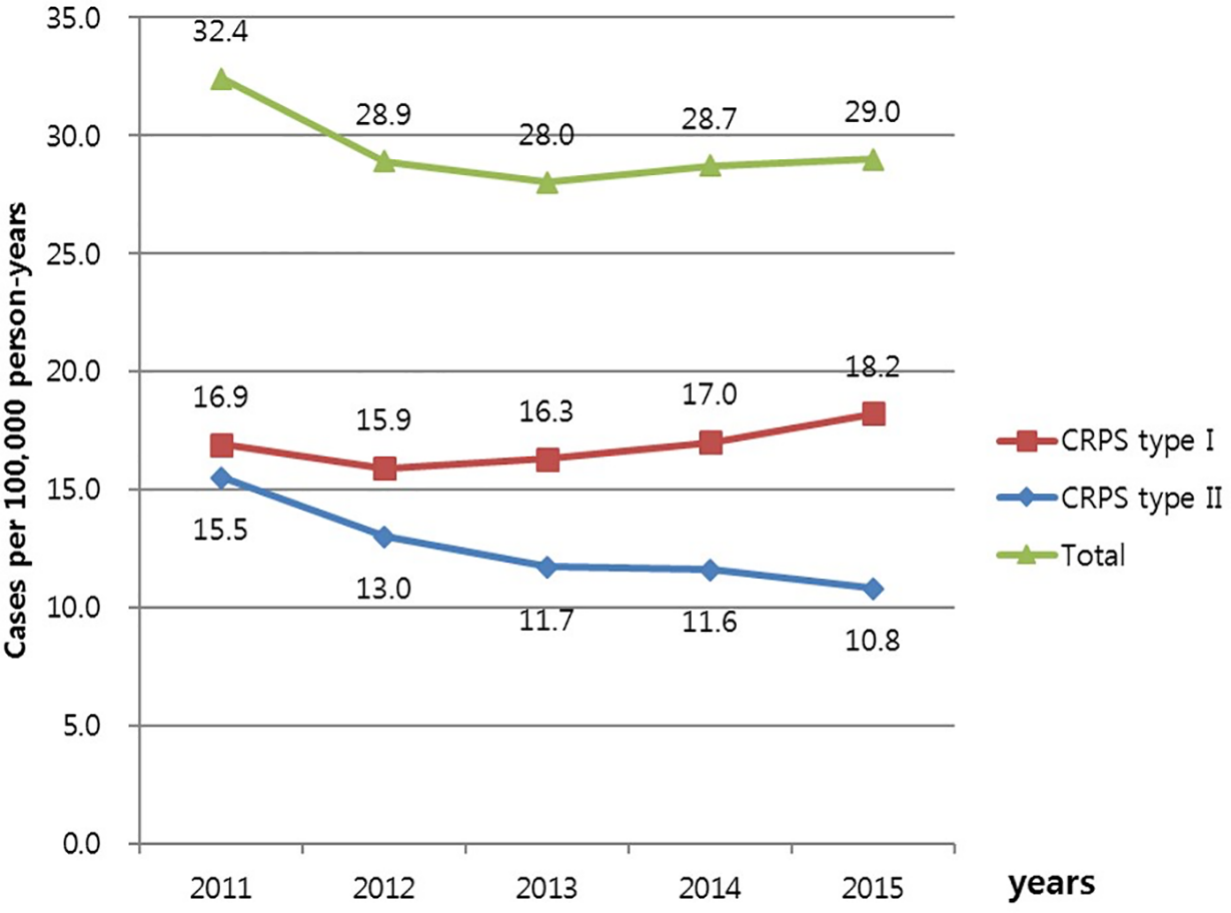
Incidence per 100,000 person-years from 2011 to 2015.

The total number of patients with CRPS from 2011 to 2015 was 74,349, resulting in the overall incidence rate of 28.0–32.4 per 100,000 person-years; among these, the number of patients with CRPS type I was 31,645 (18.2 per 100,000 person-years), and the number of patients with CRPS type II was 42,704 (10.8 per 100,000 person-years). There was no significant change in the annual number of patients from 2011 to 2015, but the incidence of CRPS type I was increased while that of CRPS type II was decreased each year (Fig 1).

### 2. Invaded part of CRPS type I in 2015

**Fig 2:**
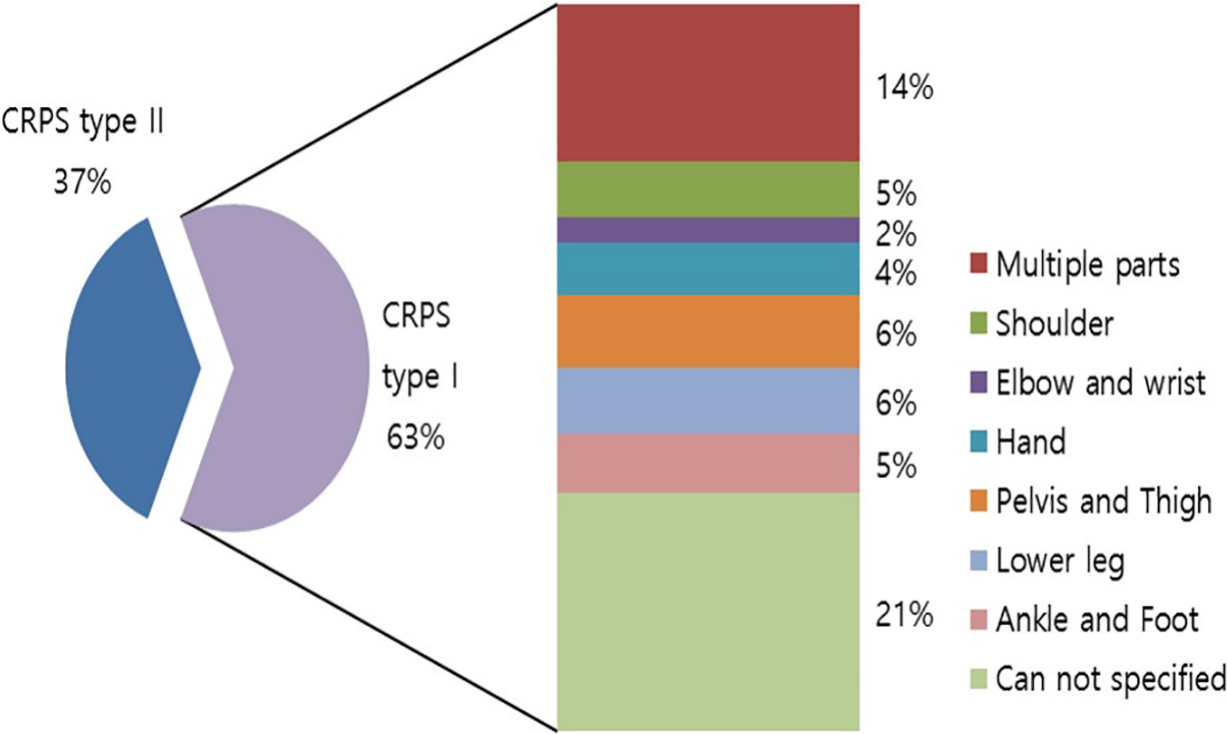
The involved body part in patients with CRPS in 2015.

Of the diagnosed cases of CRPS, the majority (63%) were CRPS type I compared with CRPS type II (37%) in 2015. The commonly involved body-parts of patients with CRPS type I were pelvis and thigh (6%) and lower leg (6%) as a single part, while multiple-part involvement including more than two symptomatic regions (14%) was more common than any other single body-part (Fig 2).

### 3. Distribution of CRPS by age group and sex in 2015

**Fig 3:**
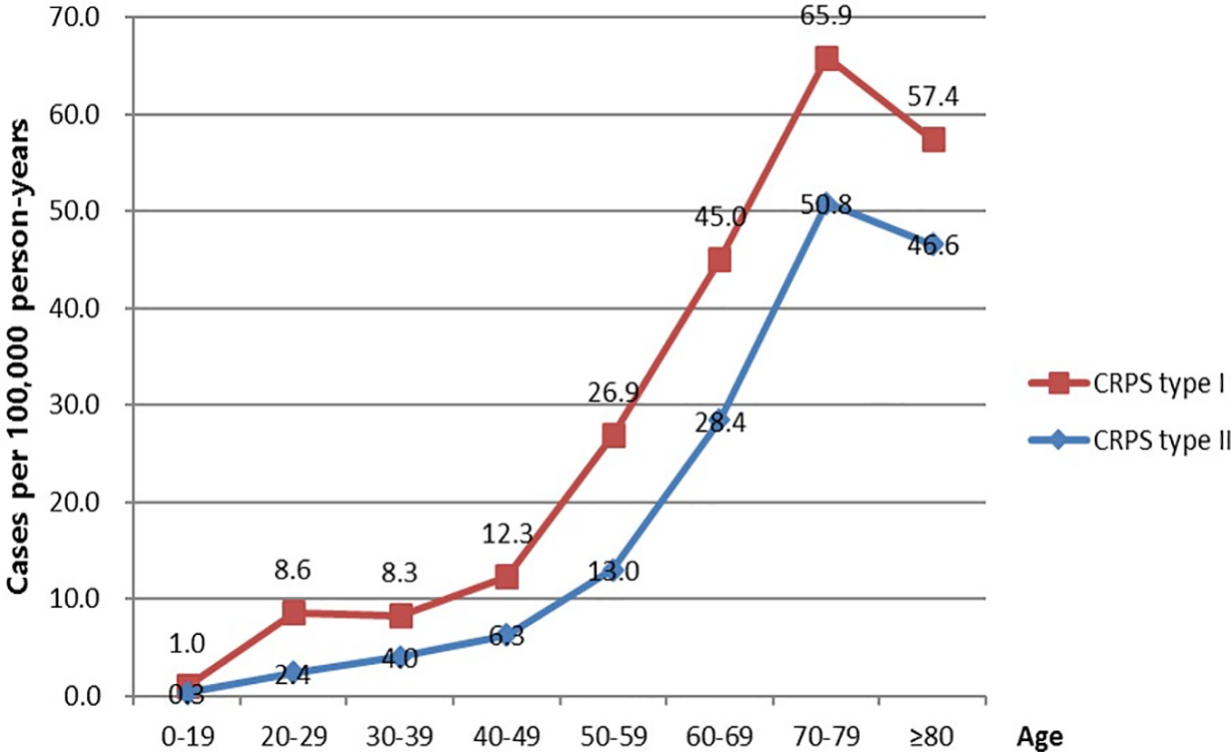
Distribution of CRPS by patient age group in 2015.

**Fig 4:**
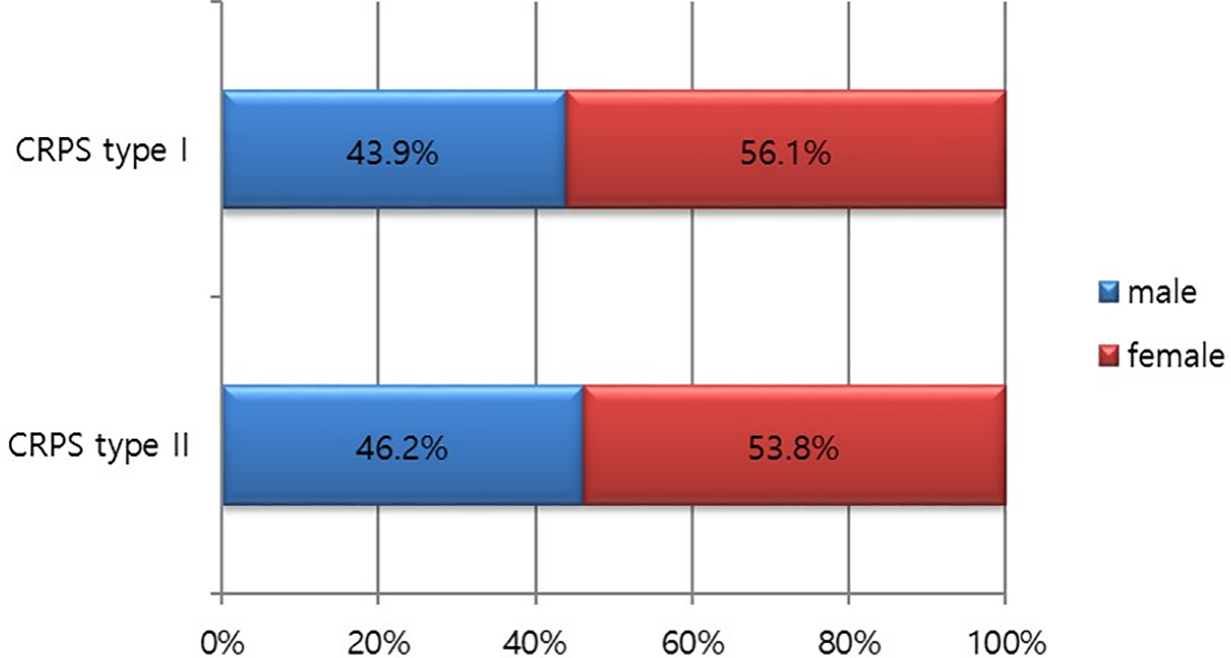
Distribution of CRPS by patient sex in 2015.

The incidence of CRPS was correlated with the patients’ age. More than half of the patients were aged over 50 years. The incidence rate of patients in the 70s age group (65.9 per 100,000 person-years) was higher than that in any of the other age groups, although the number of patients with CRPS was the highest in the 50s age group. In patients aged over 80 years, the incidence of CRPS decreased (Fig 3). Women showed a higher prevalence of CRPS than men in both CRPS type I and II. The incidence was 10.2 versus 8.0 per 100,000 person-years for women and men, respectively, in CRPS type I. The difference of prevalence of CRPS type I between male and female patients was greater than that of CRPS type II (Fig 4, Table 1).

**Table 1:** Incidence of CRPS by patient sex in 2015.

	CRPS type I	CRPS type II
Incidence per 100,000 person-year (male)	8.0	5.0
Incidence per 100,000 person-year (female)	10.2	5.8

### 4. Seasonal variation of the occurrence of CRPS in 2015

**Fig 5:**
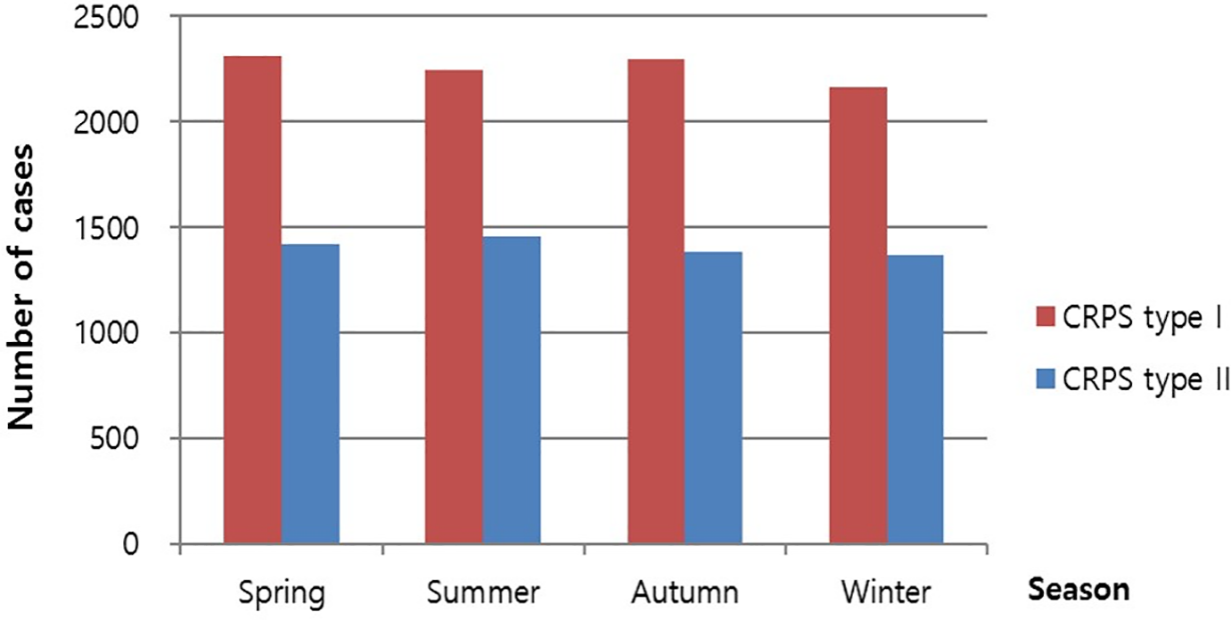
Distribution of CRPS by seasons in 2015.

CRPS cases were categorized according to the season of disease occurrence. In CRPS type I, of all CRPS cases, 25.6%, 24.8%, 25.4%, 24.0% occurred in spring (2,310 in March, April, and May), summer (2,240 in June, July, and August), fall (2,294 in September, October, and November), and winter (2,167 in December, January, and February), respectively. A trend in the seasonal variation in the occurrence of CRPS was not observed from month to month in 2015 (Fig 5).

### 5. Regional distribution of CRPS in 2015

**Fig 6:**
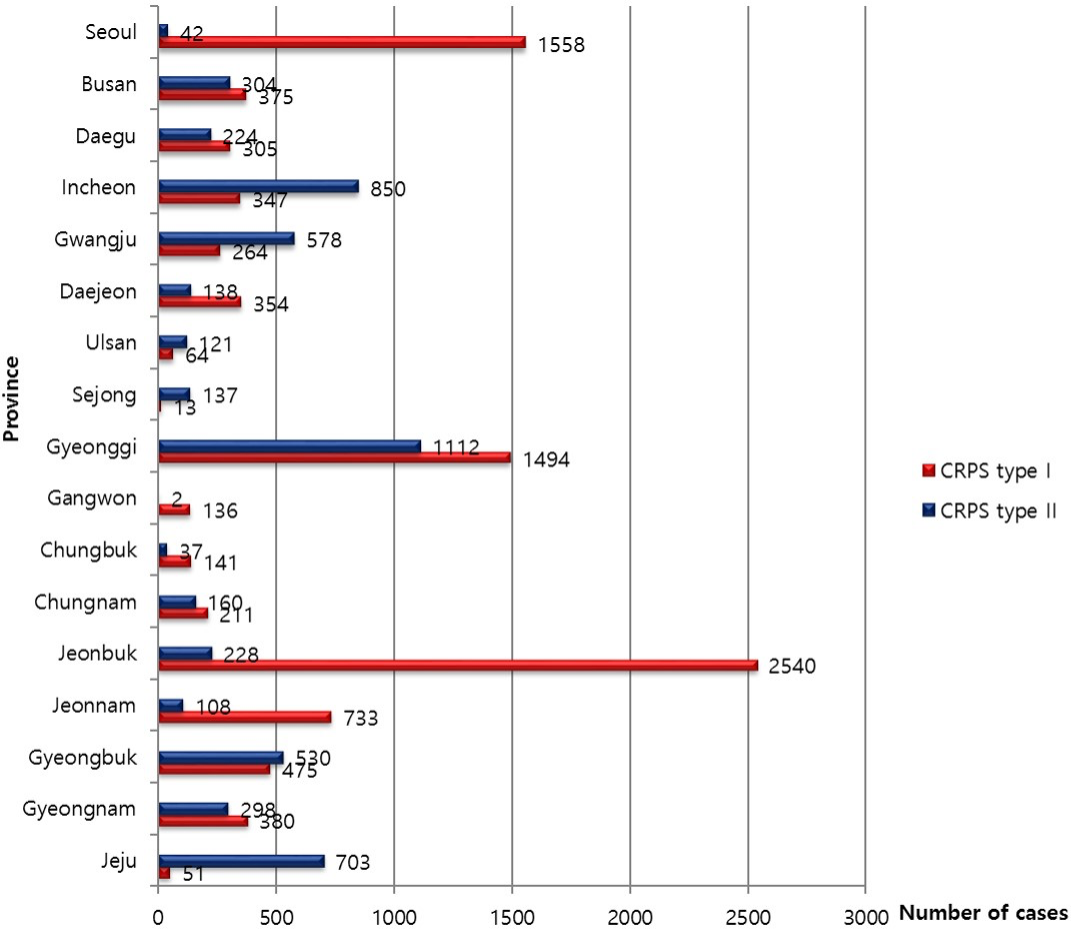
Regional (Provincial) distribution of CRPS types I and II in 2015.

The population density of each province was considered to calculate the relative rate of the occurrence of CRPS. The incidence of CRPS type I and type II among the provinces was highly variable by the province in 2015. CRPS type I was more frequent than CRPS type II in the provinces of Jeonbuk, Jeonnam, and Seoul; however, CRPS type II was more prevalent than CRPS type I in the provinces of Incheon, Jeju, and Incheon according to the number of patients (Fig 6).

### 6. Medical specialty associated with the case management for CRPS in 2015

**Fig 7:**
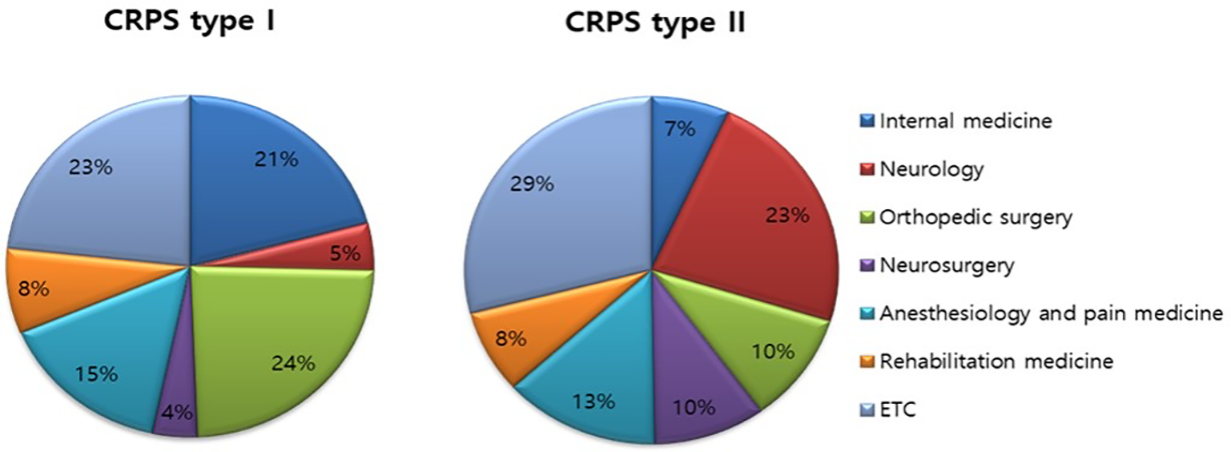
Medical specialty associated with the management of patients with CRPS in 2015.

**Fig 8:**
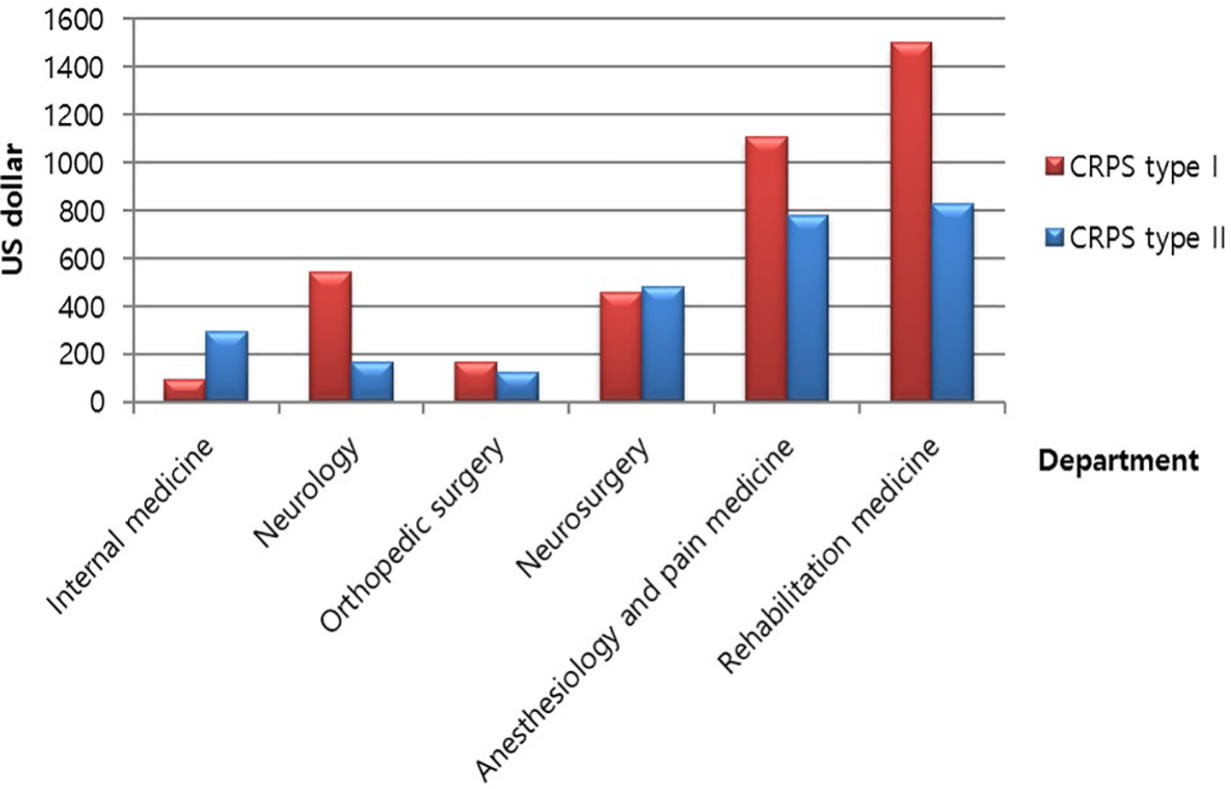
Average medical cost per visit by the medical specialty in 2015.

Based on the NHIS data, patients with CRPS type I generally visited orthopedic surgeons (24%), internal medicine physicians (21%), and anesthesiologists (15%), in order, in 2013. However, patients with CRPS type II visited the neurologist (23%) and anesthesiologists (13%) most, in order, in 2013 (Fig 7). The average medical cost per visit for CRPS II was more than 800 USD (average exchange rate in 2015: 1 USD = 1107 Korean won) in rehabilitation medicine; however, the cost was less than 800 USD in other medical specialties. The medical cost for treatment of patients with CRPS I was higher than the cost for treatment of patients with CRPS II. Patients with CRPS I spent more than 1,500 USD in rehabilitation medicine, which was the highest cost among all medical specialties. In addition, the patients spent more than 1,000 USD in anesthesiology, and less than 600 USD in other specialties (Fig 8).

## Discussion

To our best knowledge, this is the first epidemiological study on CRPS in Korea based on the largest electronic population data. Little published data are available on the epidemiology of CRPS in Korea. The previously reported results from a retrospective single-center study based on a chart review of 150 patients with CRPS are not representative of the characteristics of CRPS in Korea [6].

Based on the results from our study, the incidence of CRPS in Korea was 29.0 per 100,000 person-years with a peak incidence at the patient age of 70–79 years. This incidence was higher than that of CRPS in western countries. Previous population-based studies of CRPS showed a lower incidence compared to our current finding i.e., 26.2 per 100,000 person-years in the Netherlands,^5^ and 20.57 per 100,000 person-years in the US [7]. The incidence rate of CRPS increased with age. The highest incidence of CRPS (65.9 per 100,000 person-years) occurred in patients in the 70s age group, although the number of patients with CRPS was the highest in the 50s age group. The peak age for the occurrence of CRPS was 70–79 years, which was higher than that reported in previous studies (61–70 years and 37–50 years, respectively) [5], [8].

CRPS type I occurred more frequently in women (20.3 per 100,000 person-years) than men (16.1 per 100,000 person-years), but there was no significant difference in the frequency of occurrence of CRPS type II in both genders. Zollinger et al [9], noted that postmenopausal women have a higher prevalence rate due to hormonal etiological factors. With regards to the cultural background, women in the elderly population generally tend to receive more medical care in Korea, which could have affected the result [10], [11]. The male to female ratio in our study was 1:1.3, which differed from the findings of other studies. Many previous studies reported sex ratios of 1:2.3–4.5 [7], [12], [13]. This discrepancy may be due to ethnicity, socio-economic factors, and different diagnostic criteria used, because the previous studies were conducted in western countries. The occurrence of CRPS did not show significant variation by season. Regional distribution by the province was significantly different, due to unknown reasons. The diagnostic criteria for patients with CRPS is still not standardized among Korean doctors. Knowledge of standardized diagnostic guidelines are required to reduce the regional variability in the management of CRPS in Korea.

Our results showed an increasing trend in the occurrence of CRPS type I, while the occurrence of CRPS type II decreased from 2011 to 2015. The prevalence of CRPS type I was almost two-fold higher than that of CRPS type II, which was in agreement with previous studies that showed that the incidence of CRPS II in case of peripheral nerve injury varied from 2% to 14% [7]. The frequency of involvement of multiple parts was 14%, and the involvement of a single part was more common for CRPS I (86%). The pelvis and thigh, and lower limbs were the two most commonly involved single body-parts among patients with CRPS type I. Fracture and surgery are often regarded as the primary precipitating events of CRPS [7]; hence, the involvement of vulnerable fracture sites in older people such as the pelvis and lower limb showed relatively high prevalence. The results indicated that the lower limb was affected more often than the upper limb, in agreement with the finding of Allen et al [12].

The involvement of a relatively high number of multiple parts and other parts in patients with CRPS I also reflects a lack of understanding of the exact diagnostic criteria of CRPS among clinicians in Korea. According to our data on body-part involvement, the lower limb involvement was more prevalent than that of the upper limb in Korean patients, whereas, Sandroni reported that CRPS was more frequent in the upper arm [8], but bilateral involvement could be observed in early manifestation of the disorder [6]. The bilateral involvement may explain the high number of multiple parts. With regards to a new diagnosis of CRPS, changes in social perceptions of pain disorders and industrial development has resulted in the requirement of legal and objective evidence to identify causal relationships in medical legal disputes. Therefore, the rigorous application of diagnostic criteria for resolving legal judgment problems can affect the number of diagnosed cases by missing the final diagnosis of patients with actual symptoms.

In Korea, the Persistent Disability Assessment Guidelines published by the American Medical Association (AMA) are used instead of the IASP diagnostic criteria for diagnosing legal disabilities of patients with CRPS due to industrial accidents [14]. The critical pitfall of these CRPS diagnostic criteria is that the focus is on objective and physical findings rather than the patients’ subjective symptoms, because these criteria are primarily aimed at diagnosing the patients’ disability for working. As a result, the final number of patients who are diagnosed with CRPS may be inaccurately reduced [15].

Most traumatic injuries were managed in the department of orthopedics; however, our results indicated that an effective multimodal approach involving various medical departments was a challenge, which could impact early diagnosis and intervention in Korean patients with CRPS. The finding of a lower frequency of case management in other departments such as anesthesiology and pain medicine in this study, supported the above mentioned critical pitfall of the current clinical practice.

Currently, only a few evidence-based treatment modalities are available; thus, treatment typically involves therapies that are used in other neuropathic pain conditions [16]. The treatment of CRPS can be grouped as non-invasive and invasive treatment [17], [18]. Non-invasive treatment includes multimodal pharmacological therapy such as the use of nonsteroidal anti-inflammatory drugs, opioids, antidepressants, sodium channel-blocking agents, gabapentin, pregabalin, and steroid. Invasive treatment includes sympathetic ganglion block, intravenous regional sympatholysis, transcutaneous electrical nerve stimulation, and stimulation techniques, such as spinal cord or peripheral nerve stimulation. Non-invasive medical treatment is more popular in Korea due to its low cost and the lack of knowledge of invasive treatment. Our results indicated a higher average medical cost per visit in the departments of rehabilitation, and anesthesiology and pain medicine, wherein treatment mainly involves invasive modalities.

The poor treatment outcome may be partly attributed to the patients’ relatively severe pain and prolonged illness. For effective early management, patients with a presumptive diagnosis of CRPS are recommended for referral to specialists who use a multimodal approach [6]. In a previous report, the proposed management algorithm for CRPS emphasizes pain management and restoration of function, which is best attained in a comprehensive interdisciplinary setting through the early diagnosis [19]. Our results suggested a lack of multimodal management in more specialized medical departments in Korea. Improvements in developing a consensus opinion among medical doctors on diagnostic criteria and in increasing the current understanding of the pathophysiology of CRPS will facilitate effective clinical trials of mechanism-based treatment modalities [20].

Our study had some limitations. First, there is no consensus among physicians on management practices for patients with CRPS in Korea due to the presence of variable signs and symptoms. Although the objective tests presented negative results, CRPS can often be diagnosed based on the physician’s opinion of the patients’ subjective symptoms. Second, this study was a retrospective study using the HIRA database. Follow-up losses and diagnoses from multiple medical resources could have been included due to lack of connectivity of systems across the entire medical service; although multiple claims from one patient were excluded in this study. Third, precedent factors, associated disease, and psychologic factors were not considered because these are not accurately reported through individual survey. To address these limitations, further epidemiological studies are needed.

In conclusion, we determined the epidemiologic characteristics of patients with CRPS in Korea based on the largest electronic population database in Korea. The incidence rate of CRPS in Korea was 29.0 per 100,000 person-years, which showed correlation with age. Considering the rapidly aging general population, increasing industrialization, and diverse associated diseases, the current incidence rate of CRPS may be changed. With evidence based consensus, exact diagnostic criteria of CRPS are needed between the physician related to management of CRPS, which can avoid misdiagnosis and make start effective treatment. Further research is required to address physicians’ consensus on the definite diagnosis, and the cost-effectiveness of the therapeutic modality, in order to obtain more accurate epidemiological data on CRPS. In addition, a continuous nation-wide study on the epidemiologic characteristics of Korean patients with CRPS is required as a means of facilitating early management practices.

## Supporting information

S1 File File containing raw data of study subjects. (XLSX)

## References

[1] Bruehl S, Harden RN, Galer BS, Saltz S, Backonja M, Stanton-Hicks M. Complex regional pain syndrome: are there distinct subtypes and sequential stages of the syndrome? *Pain*. 2002; 95: 119–24. 11790474 10.1016/s0304-3959(01)00387-6

[2] Birklein F: Complex regional pain syndrome. *J Neurol*. 2005; 252: 131–8. doi: 10.1007/s00415-005-0737-8 15729516 10.1007/s00415-005-0737-8

[3] Kim YC. Complex regional pain syndrome. *Korean J Pain*. 2004;17:S104–8.

[4] Harden N, Bruehl S, Stanton-Hicks M, Wilson PR.: Proposed new diagnostic criteria for complex regional pain syndrome. *Pain Med*. 2007; 8: 326–31. doi: 10.1111/j.1526-4637.2006.00169.x 17610454 10.1111/j.1526-4637.2006.00169.x

[5] Mos D, Bruijn AGJ, Huygen FJPM, Dieleman JP, Stricker BH, Sturkenboom MCJM. The incidence of complex regional pain syndrome: a population-based study. *Pain*. 2007; 129: 12–20. doi: 10.1016/j.pain.2006.09.008 17084977 10.1016/j.pain.2006.09.008

[6] Choi YS, Lee MG, Lee HM, Lee CJ, Jo JY, Jeon SY, Lee SC, Kim YC. Epidemiology of complex regional pain syndrome: a retrospective chart review of 150 Korean patients. *J Korean Med Sci*. 2008; 23: 772–5. doi: 10.3346/jkms.2008.23.5.772 18955780 10.3346/jkms.2008.23.5.772 PMC2580014

[7] Veldman PH, Reynen HM, Arntz IE, Goris RJ. Signs and symptoms of reflex sympathetic dystrophy: prospective study of 829 patients. *Lancet*. 1993;342:1012–6. 8105263 10.1016/0140-6736(93)92877-v

[8] Sandroni P, Benrud-Larson LM, McChelland RL, Low PA. Complex regional pain syndrome type I: incidence and prevalence in Olmsted county, a population-based study. *Pain*. 2003;103:199–207. 12749974 10.1016/s0304-3959(03)00065-4

[9] Zollinger PE, Tuinebreijer WE, Kreis RW, Breederveld RS. Effect of vitamin C on frequency of reflex sympathetic dystrophy in wrist fracture: a randomized trial. *Lancet*. 1999; 354: 2025–8. doi: 10.1016/S0140-6736(99)03059-7 10636366 10.1016/S0140-6736(99)03059-7

[10] Jeon GS, Choi ES, Lee HY. Gender-related difference in the utilization of health care services by Korean adults. *J Korean Acad Public Health Nurs*. 2010; 24: 182–196.

[11] Shin SM, Kim ES, Lee HW. The contributing factors to surplus medicine by long-term users of medical aid in Korea. *J Prev Med Public Health*. 2009; 42: 403–7. doi: 10.3961/jpmph.2009.42.6.403 20009487 10.3961/jpmph.2009.42.6.403

[12] Allen G, Galler BS, Schwartz L. Epidemiology of complex regional pain syndrome type I: a retrospective chart review of 134 patients. *Pain*. 1999; 80: 539–44. 10342415 10.1016/S0304-3959(98)00246-2

[13] Stanton-Hicks M, Janig W, Hassenbusch S, Haddox JD, Boas R, Wilson P. Reflex sympathetic dystrophy: changing concepts and taxonomy. *Pain*. 1995; 63: 127–33. 8577483 10.1016/0304-3959(95)00110-E

[14] Cocchiarella L, Andersson GB: Guides to the Evaluation of Permanent Impairment 5th ed Chicago, AMA press 2001, 336–7.

[15] Kim YC: The evaluation of permanent impairment in chronic pain patients. *Korean J Pain*. 2007; 20: 1–7.

[16] Tran DH, Duong S, Bertini P, Finlayson RJ. Treatment of complex regional pain syndrome. a review of the evidence. *Can J Anesth*. 2010; 57: 149–66. doi: 10.1007/s12630-009-9237-0 20054678 10.1007/s12630-009-9237-0

[17] Schwartzman RJ, Kerrigan J. The movement disorder of reflex sympathetic dystrophy. *Neurology*. 1990; 40: 57–61. 2296383 10.1212/wnl.40.1.57

[18] Stanton-Hicks MD, Baron R, Boas R, Gordh T, Harden N, Hendler N, et al Complex regional pain syndromes: guidelines for therapy. *Clinical Journal of Pain*. 1998; 14: 155–66. 9647459 10.1097/00002508-199806000-00012

[19] Stanton-Hicks MD, Burton AW, Bruehl SP, Carr DB, Harden RN, Hassenbusch SJ, et al An updated interdisciplinary clinical pathway for CRPS: report of an expert panel. *Pain Practice*. 2002; 2: 1–16. doi: 10.1046/j.1533-2500.2002.02009.x 17134466 10.1046/j.1533-2500.2002.02009.x

[20] Coderre TJ, Xanthos DN, Francis L, Bennett GJ. Chronic post-ischemia pain (CPIP). a novel animal model of complex regional pain syndrome-Type I (CRPS-I; reflex sympathetic dystrophy) produced by prolonged hindpaw ischemia and reperfusion in the rat. *Pain*. 2004; 112: 94–105. doi: 10.1016/j.pain.2004.08.001 15494189 10.1016/j.pain.2004.08.001

