# Correction: Epidemiology of complex regional pain syndrome in Korea: An electronic population health data study

**DOI:** 10.1371/journal.pone.0201327

**Published:** 2018-07-23

**Authors:** The PLOS ONE Staff

There is an error in the first sentence of the “1. Incidence of CRPS from 2011 to 2015” section in the Results. The correct sentence is: The total number of patients with CRPS from 2011 to 2015 was 74,349, resulting in the overall incidence rate of 28.0–32.4 per 100,000 person-years; among these, the number of patients with CRPS type I was 42,704 (18.2 per 100,000 person-years), and the number of patients with CRPS type II was 31,645 (10.8 per 100,000 person-years).

S1 File is an untranslated file. The publisher apologizes for the error. Please view the translated S1 File below.

## Supporting information

S1 File File containing raw data of study subjects. (XLSX)
